# Investigating the impact of metabolic syndrome traits on telomere length: a Mendelian randomization study

**DOI:** 10.1002/oby.23810

**Published:** 2023-07-06

**Authors:** Nellie Y. Loh, Daniel Rosoff, Raymond Noordam, Constantinos Christodoulides

**Affiliations:** ^1^ Oxford Centre for Diabetes, Endocrinology and Metabolism, Radcliffe Department of Medicine University of Oxford Oxford UK; ^2^ Section on Clinical Genomics and Experimental Therapeutics National Institute on Alcohol Abuse and Alcoholism, National Institutes of Health Bethesda Maryland USA; ^3^ Department of Internal Medicine, Section of Gerontology and Geriatrics Leiden University Medical Center Leiden the Netherlands; ^4^ National Institute for Health Research, Oxford Biomedical Research Centre Oxford University Hospitals National Health Service Foundation Trust Oxford UK

## Abstract

**Objective:**

Observational studies have reported bidirectional associations between metabolic syndrome (MetS) traits and short leukocyte telomere length (LTL), a TL marker in somatic tissues and a proposed risk factor for age‐related degenerative diseases. However, in Mendelian randomization studies, longer LTL has been paradoxically associated with higher MetS risk. This study investigated the hypothesis that shorter LTL might be a consequence of metabolic dysfunction.

**Methods:**

This study undertook univariable and multivariable Mendelian randomization. As instrumental variables for MetS traits, all of the genome‐wide significant independent signals identified in genome‐wide association studies for anthropometric, glycemic, lipid, and blood pressure traits conducted in European individuals were used. Summary‐level data for LTL were obtained from a genome‐wide association study conducted in the UK Biobank.

**Results:**

Higher BMI was associated with shorter LTL (*β* = −0.039, 95% CI: −0.058 to −0.020, *p* = 5 × 10^−5^) equivalent to 1.70 years of age‐related LTL change. In contrast, higher low‐density lipoprotein cholesterol was associated with longer LTL (*β* = 0.022, 95% CI: 0.007 to 0.037, *p* = 0.003) equivalent to 0.96 years of age‐related LTL change. Mechanistically, increased low‐grade systemic inflammation, as measured by circulating C‐reactive protein, and lower circulating linoleic acid levels might link higher BMI to shorter LTL.

**Conclusions:**

Overweight and obesity might promote the development of aging‐related degenerative diseases by accelerating telomere shortening.

**Study Importance**

**What is already known?**

- Cross‐sectional and longitudinal observational studies have reported bidirectional associations between metabolic syndrome traits and short leukocyte telomere length (LTL), a proposed risk factor for age‐related degenerative diseases.
- Using two‐sample Mendelian randomization, we previously demonstrated that longer LTL was paradoxically associated with higher metabolic syndrome risk.

**What does this study add?**

- Higher BMI is associated with shorter LTL, equivalent to approximately 1.7 years of age‐related LTL change.
- Higher low‐density lipoprotein cholesterol is associated with longer LTL.
- Increased subclinical inflammation and lower circulating linoleic acid levels might link obesity to shorter LTL.

## INTRODUCTION

The metabolic syndrome (MetS) is a constellation of interrelated factors that increase the risk for type 2 diabetes and atherosclerosis [1]. These factors include hyperglycemia, hypertension, dyslipidemia marked by raised triglycerides (TAG) and low high‐density lipoprotein cholesterol (HDL‐C), and abdominal obesity. Regardless of the definition used, the global prevalence of MetS is high and rising [2].

Telomeres are complexes of repetitive DNA sequences and specialized proteins that cap the ends of eukaryotic chromosomes and function to maintain chromosomal integrity and stability. In proliferating cells, telomeres shorten with each cell division. Shortened telomeres ultimately reach a critical length, which results in DNA damage with ensuing loss of replicative capacity and cell senescence or apoptosis. This, in turn, leads to compromised stem and/or progenitor cell function, tissue atrophy, and functional decline [3, 4]. Telomere length (TL) differs considerably among individuals and it is genetically determined, with heritability estimates between 44% and 86% [5]. Accelerated telomere attrition is also thought to result from increased exposure to oxidative stress and chronic low‐grade inflammation, which are considered important drivers of biological aging [6, 7].

TL within individuals is generally strongly correlated across tissues [8] and, consequently, is typically measured in leukocytes because of their easy accessibility in peripheral blood. Shorter leukocyte TL (LTL) has been associated with older age [9], lower life expectancy [10, 11], and higher risk of coronary artery disease [11, 12]. Several epidemiological studies have also highlighted bidirectional associations between LTL and MetS traits. Specifically, short LTL was reported to be associated with the prevalence [13, 14] and progression [13] of multiple components of MetS. Conversely, the presence of MetS traits was shown to be associated with both lower LTL and higher LTL attrition [15, 16, 17, 18]. However, aside from the links between adiposity traits and LTL, the last findings were not replicated in the largest cross‐sectional study investigating these associations in 422,797 UK Biobank (UKBB) participants [19]. Furthermore, results from observational studies cannot be used to infer causality because they might be subject to residual confounding and/or reverse causation bias. Additionally, because of cost and practical implications, epidemiological studies cannot, through serial MetS trait and LTL measurements, determine the consequences of lifetime exposure to MetS or its components on LTL attrition. Consequently, whether MetS traits are a cause or consequence or indeed are causally linked with short LTL remains unknown.

Mendelian randomization (MR) is an epidemiological tool using data from genetic studies to estimate the nonconfounded associations between exposures and outcomes. We and others have recently explored the relationships between LTL and MetS traits using MR but failed to detect any causal associations between short telomeres and higher risk of MetS or its constituent traits [11, 20]. On the contrary, longer LTL was associated with higher blood pressure (BP), higher waist‐hip ratio adjusted for body mass index (WHRadjBMI), and increased MetS risk [20]. Prompted by these paradoxical findings, we hypothesized that shorter LTL might be a consequence of metabolic dysfunction.

## METHODS

We investigated the associations of MetS and MetS traits with LTL in men and women of European descent using MR. As exposure instruments, we used all genome‐wide significant (*p* < 5 × 10^−8^), independent single‐nucleotide polymorphisms (SNPs) identified in genome‐wide association study (GWAS) meta‐analyses for anthropometric (BMI and WHRadjBMI), glycemic (fasting glucose and insulin), lipid (TAG, HDL‐C, and low‐density lipoprotein cholesterol [LDL‐C]), and BP traits, with MetS as a binary trait, conducted in European individuals pruned for linkage disequilibrium (LD) using ld_clump, with the default set at LD *r* ^2^ < 0.001 and a genetic distance of 10 megabases (Mb). In the case of fasting insulin, because of the small number of identified sex‐specific GWAS‐significant SNPs (3 for women, 2 for men), we used the 19 fasting insulin SNPs established by the Meta‐Analyses of Glucose and Insulin‐related traits Consortium (MAGIC) [21] as instrumental variables (IVs). Where GWAS summary statistics for MetS traits were not publicly available, IVs were obtained from a GWAS that we conducted in the UKBB (Supporting Information Table S1).

As outcome data for the sex‐combined analyses, we used summary‐level data for LTL from a GWAS conducted in the UKBB (ieu‐b‐4879) [11] and, for the sex‐specific studies, summary‐level data from a newly conducted GWAS, also in the UKBB.

To examine reverse causality, we conducted MR analyses using IVs extracted from the LTL GWAS using extract_instruments() in the TwoSampleMR package (version 0.5.6) and outcome data using GWAS summary statistics for MetS and MetS traits from the aforementioned studies.

We used Functional Mapping and Annotation of Genome–Wide Association Studies (FUMA) (https://fuma.ctglab.nl/) [22] to explore potential mechanisms in which BMI and LDL‐C affect LTL. We used the SNP2GENE function of FUMA to translate GWAS signals for BMI (GCST009004) and LDL‐C (GCST002222) into sets of mapped genes. We then used the GENE2FUNCTION tool in FUMA, which performs hypergeometric tests, to test whether genes of interest are overrepresented in predefined pathways, i.e., in this instance, reported genes from the GWAS catalog (www.ebi.ac.uk/gwas/). Finally, we cross‐referenced the GENE2FUNCTION gene set output with traits and indices of healthy behavior previously shown to be cross‐sectionally associated with LTL [19, 23]. The latter, which are highlighted in Supporting Information Tables S2 and S3, were taken forward for further MR analyses, including mediation studies with multivariable MR (MVMR). MR analyses were performed using publicly available European population‐specific GWAS summary statistics for omega‐3 and omega‐6 fatty acids (FAs) [24], linoleic acid (LA) [24], years of schooling [25], and, in the case of C‐reactive protein (CRP), summary statistics from a GWAS that we conducted in the UKBB. Finally, in cases in which exposure and outcome data were derived from the same or overlapping populations, additional MR analyses were performed using European population‐specific summary data from independent cohorts (online Supporting Information Methods, Figure S1 and Table S1).

We used the inverse‐variance weighted (IVW) approach for MR analyses, with MR‐Egger regression, weighted–median MR, and contamination mixture (Conmix) MR as sensitivity analyses. Additional sensitivity analyses for exposures with UKBB participants included calculating the variability in instrument strength (*I* ^2^) to assess bias for the MR‐Egger method [26] and the MRlap method, which was recently developed to account for sample overlap and which also assesses weak instrument bias and winner's curse, for exposure‐outcome pairs with sample overlap [27]. We also performed colocalization [28] and cis‐instrument MR assessing the role of the FA desaturase (*FADS*) gene region in the polyunsaturated FA (PUFA)‐LTL relationships (online Supporting Information Methods). Furthermore, we conducted MR analyses after excluding variants with larger effects on outcome than the exposure trait (Steiger filtering). Results were corrected for multiple testing, with *p* < 0.0125 (0.05/4) considered significant. This cutoff is based on assessments of the associations of four classes of MetS traits (anthropometric, glycemic, lipid, and BP) with LTL. A statistically significant IVW result coupled with directionally consistent associations from all three sensitivity analyses was considered sufficient evidence to claim a causal effect.

MVMR analyses were undertaken only if the primary MR analyses involving the MetS trait, mediator of interest, and LTL outcome were significant after Bonferroni correction, with directionally consistent associations in all three sensitivity analyses. For this, we used GWAS summary statistics derived from nonoverlapping or, in the case of BMI, partially overlapping samples for exposure and outcome data. Exposure data for MetS trait and mediator of interest were extracted for MVMR using mv_extract_exposures(). MVMR was subsequently performed to estimate the direct effect of the MetS trait when adjusted for the effect of the mediator and vice versa. In MVMR analyses involving the omega‐6 FA and LA UKBB GWAS, the UKBB cohort was partitioned to exclude the 114,999 UKBB participants in the omega‐6 FA and LA GWAS and a new LTL GWAS generated to minimize bias due to sample overlap.

All MR and MVMR analyses were conducted using the TwoSampleMR (version 0.5.6) and MendelianRandomization (version 0.6.0) packages in R (version 4.2.1) [29].

## RESULTS

### Associations between MetS traits and LTL

To estimate the associations of MetS and its component traits with LTL, we conducted one‐ and two‐sample MR. As IVs for MetS traits, we used all of the genome‐wide significant independent signals identified in GWAS meta‐analyses for anthropometric (up to 694,649 participants), glycemic (up to 151,188 participants), lipid (up to 188,577 participants), and BP (up to 757,601 participants) traits conducted in European individuals (Supporting Information Table S1). Additionally, we selected 93 genome‐wide significant independent variants from a GWAS conducted in the UKBB (291,107 participants) as IVs for MetS as a binary trait. Summary‐level data for LTL for the sex‐combined analyses were obtained from a GWAS conducted in the UKBB (472,174 participants) [11] or generated from a newly conducted GWAS, also in the UKBB, for the sex‐specific studies (241,110 women and 203,071 men).

**FIGURE 1:**
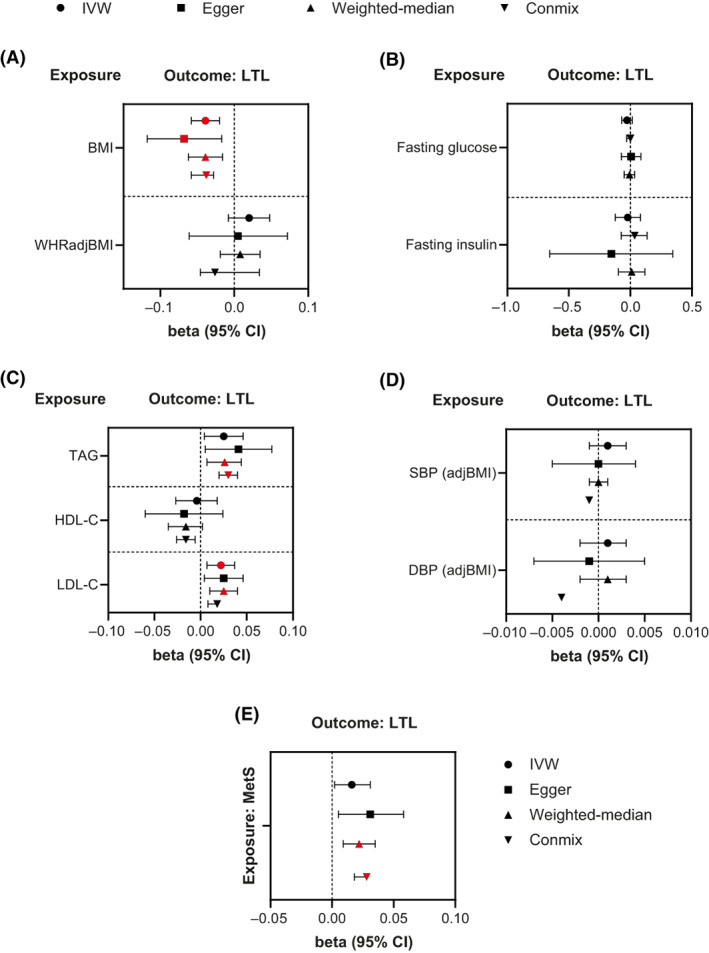
MR estimates of the effects of MetS traits and MetS as a binary trait on LTL in sex‐combined European populations. IVW, MR‐Egger, weighted‐median, and Conmix estimates (with 95% CI) from all the primary MR analyses are shown for the effects of (A) anthropometric, (B) glycemic, (C) lipid, and (D) BP traits and (E) MetS as a binary trait on LTL (Supporting Information Table S4). Red‐filled symbols: *p* < 0.0125 (Bonferroni correction for multiple testing). IVW *p* < 0.0125 coupled with directionally consistent results across all sensitivity analyses was considered as sufficient evidence to claim a causal effect. BP, blood pressure; Conmix, contamination mixture; DBP (adjBMI), BMI‐adjusted diastolic BP; HDL‐C, high‐density lipoprotein cholesterol; IVW, inverse‐variance weighted; LDL‐C, low‐density lipoprotein cholesterol; LTL, leukocyte telomere length; MetS, metabolic syndrome; MR, Mendelian randomization; SBP (adjBMI), BMI‐adjusted systolic BP; TAG, triglycerides; WHRadjBMI, waist‐hip ratio adjusted for BMI [Color figure can be viewed at wileyonlinelibrary.com]

Using IVW MR, higher BMI and elevated LDL‐C were associated with shorter (*β* = −0.039, 95% confidence interval [CI]: −0.058 to −0.020, *p* = 5 × 10^−5^) and longer (*β* = 0.022, 95% CI: 0.007 to 0.037, *p* = 0.003) LTL, respectively (Figure 1, Supporting Information Table S4). Nominally positive associations were also detected between raised TAG and MetS as a binary trait and LTL (Supporting Information Table S4). The association between BMI and shorter LTL was confirmed and extended by demonstrating that body fat percentage was also inversely associated with LTL (*β* = −0.074, 95% CI: −0.102 to −0.045, *p* = 5 × 10^−7^). However, no significant associations of favorable or unfavorable adiposity with LTL were detected (Supporting Information Table S5). In sex‐specific analyses, higher BMI appeared to be more strongly associated with shorter LTL in women, whereas the associations of LDL‐C and TAG with LTL appeared to be stronger in men. However, the *p* values for the test of interaction by sex for these analyses were nonsignificant (Supporting Information Table S6).

To detect violations of MR assumptions, we conducted sensitivity analyses using MR‐Egger, weighted‐median MR, and Conmix MR (Figure 1, Supporting Information Table S4). All methods yielded directionally consistent associations with IVW. Furthermore, the MR‐Egger intercept did not detect evidence of directional pleiotropy for any of the significant outcomes. Similarly, MR analyses using Steiger‐filtered genetic instruments yielded consistent findings (Supporting Information Table S4). Because the IVs for the analyses involving BMI, body fat percentage, and LTL were derived from partially and completely overlapping populations, respectively, which may lead to biased estimates, we also conducted MR analyses in nonoverlapping samples, which generated directionally consistent IVW estimates with those obtained in the primary analyses (Supporting Information Tables S5 and S7). We further estimated the bias and type I error rate for all significant associations derived from overlapping populations [30], which were negligible, and instruments with sample overlap demonstrated high instrument strength variability (average *I* ^2^ = 98.7%, minimum *I* ^2^ = 98%; Supporting Information Table S8A), suggesting little bias for the MR‐Egger estimates in these analyses [26]. MRlap analyses evaluating sample overlap, winner's curse, and weak instrument bias [27] yielded statistically significant and directionally consistent results with the primary IVW estimates (Supporting Information Table S8B), suggesting that the latter were minimally affected by these sources of bias.

Finally, we conducted reverse MR studies to investigate the associations between LTL and MetS traits. These highlighted positive associations between LTL and WHRadjBMI (*β* = 0.045, 95% CI: 0.011–0.079, *p* = 0.009), TAG (*β* = 0.082, 95% CI: 0.032–0.133, *p* = 0.001), systolic BP (*β* = 0.053, 95% CI: 0.018–0.088, *p* = 0.003), and MetS as a binary trait (*β* = 0.106, 95% CI: 0.025–0.188, *p* = 0.01), which were both directionally consistent in sensitivity analyses and robust to Steiger filtering (Supporting Information Table S9). These results are consistent with previously published reports both from our group, using IVs for LTL derived from a smaller GWAS [20], and others [11].

### Mechanisms linking BMI and LDL‐C with altered LTL

To gain mechanistic insights into the causal associations of BMI and LDL‐C with LTL, we first performed functional gene mapping of BMI‐ and LDL‐C‐associated GWAS signals using the SNP2GENE function of FUMA (Supporting Information Figure S1) [22]. Subsequently, we used the GENE2FUNCTION tool in FUMA to test whether the sets of mapped genes associated with BMI and LDL‐C are overrepresented within sets of genes mapping to signals for other GWAS catalog traits (www.ebi.ac.uk/gwas/). Finally, we cross‐referenced the GENE2FUNCTION gene set output with traits and indices of healthy behaviors previously shown to be cross‐sectionally associated with LTL (Supporting Information Tables S2 and S3) [19, 23]. Based on these results, we postulated that subclinical inflammation, as measured by circulating CRP [19] and omega‐6 [23] and omega‐3 FAs [19], and educational attainment [19] might mediate the links of higher BMI and/or hypercholesterolemia with altered LTL.

**FIGURE 2:**
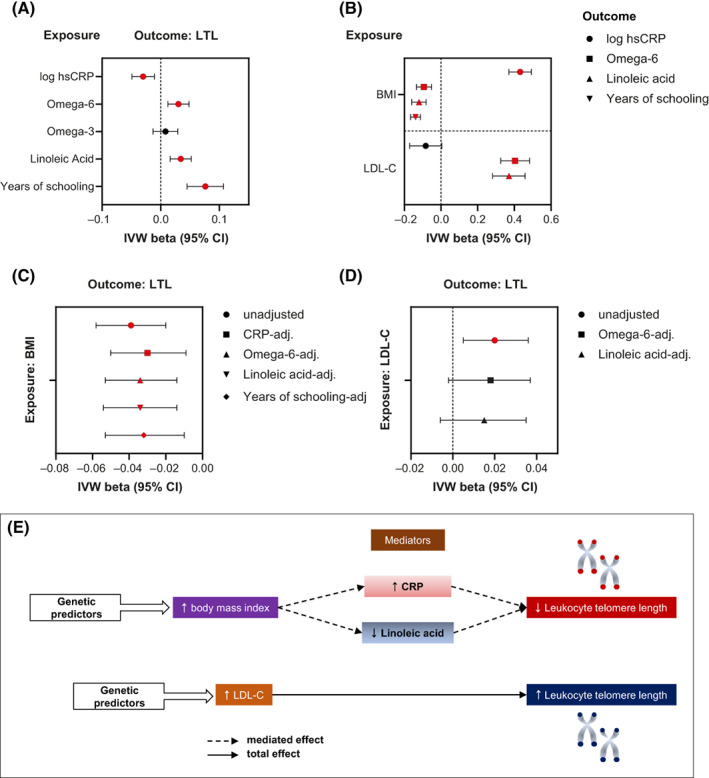
MR and MVMR investigating CRP, omega‐6 and omega‐3 FAs, LA, and years of schooling as mediators of the effects of MetS traits on LTL in sex‐combined European populations. IVW estimates (with 95% CI) from the primary MR analyses are shown for the effects of (A) CRP, omega‐6 and omega‐3 FAs, LA, and years of schooling on LTL (Supporting Information Table S10); and (B) BMI and LDL‐C on CRP, omega‐6 FAs, LA, and years of schooling (Supporting Information Table S12). Mediation analyses: IVW estimates of effects of (C) BMI and (D) LDL‐C on LTL adjusted for indicated mediator (Supporting Information Table S15). Red‐filled symbols: Analyses yielding IVW results with *p* < 0.0125 (Bonferroni correction for multiple testing) and that are directionally consistent across all sensitivity analyses (panels A and B) and *p* < 0.05 with adjustment for mediator (panels C and D). (E) Summary findings of MR and MVMR analyses. CRP, C‐reactive protein; FA, fatty acid; hsCRP, high sensitivity CRP; IVW, inverse‐variance weighted; LA, linoleic acid; LDL‐C, low‐density lipoprotein cholesterol; LTL, leukocyte telomere length; MetS, metabolic syndrome; MR, Mendelian randomization; MVMR, multivariable MR [Color figure can be viewed at wileyonlinelibrary.com]

Next, we investigated the associations between the aforementioned traits and LTL. As exposure instruments, we used all of the genome‐wide significant independent signals identified from the following: 1) a GWAS of CRP that we newly conducted in the UKBB; 2) the largest GWAS of the plasma metabolome, also conducted in the UKBB (up to 115,078 participants) [24]; and 3) a GWAS meta‐analysis of educational attainment in 766,345 European‐descent individuals (Supporting Information Table S1) [25]. Using IVW MR, only elevated CRP (*β* = −0.030, 95% CI: −0.049 to −0.011, *p* = 0.002); omega‐6 FAs (*β* = 0.030, 95% CI: 0.012 to 0.048, *p* = 0.001); LA (*β* = 0.034, 95% CI: 0.016 to 0.052, *p* = 0.0002), the most prevalent dietary omega‐6 FA in Western diets; and years of schooling (*β* = 0.076, 95% CI: 0.045 to 0.107, *p* = 1 × 10^−6^) were associated with LTL (Figure 2A, Supporting Information Table S10). MR with cis CRP IVs did not support a direct association between CRP and LTL, consistent with CRP functioning as a surrogate for low‐grade systemic inflammation (Supporting Information Table S10). Finally, MVMR analyses provided suggestive evidence that the associations of omega‐6 FAs and LA with LTL might be independent of other PUFAs (*β* ± standard error [SE]: 0.081 ± 0.048, *p* = 0.09 for both associations after adjustment for total PUFA; Supporting Information Table S11). Additional colocalization and cis‐instrument MR analyses examining the relationships of SNPs mapping to the *FADS* locus, which are highly pleiotropic and not specific for individual FAs or FA classes, and LTL revealed that the omega‐6– and LA‐LTL relationships were independent of *FADS* variants (Supporting Information Table S5). We next examined the associations of BMI and LDL‐C with systemic CRP, omega‐6 FA and LA levels, and years of schooling (Figure 2B, Supporting Information Table S12). Higher BMI was associated with elevated CRP (*β* = 0.432, 95% CI: 0.370 to 0.493, *p* = 7 × 10^−43^) but lower omega‐6 FA (*β* = −0.093, 95% CI: −0.134 to −0.052, *p* = 1 × 10^−5^) and LA (*β* = −0.120, 95% CI: −0.160 to −0.081, *p* = 2 × 10^−9^) levels and fewer years of schooling (*β* = −0.139, 95% CI: −0.165 to −0.112, *p* = 2 × 10^−24^). In contrast, higher LDL‐C was positively associated with plasma levels of omega‐6 FAs (*β* = 0.404, 95% CI: 0.326 to 0.483, *p* = 7 × 10^−24^) and LA (*β* = 0.371, 95% CI: 0.282 to 0.459, *p* = 3 × 10^−16^) and, in Steiger‐filtered analyses, negatively associated with CRP (*β* = −0.059, 95% CI: −0.097 to −0.021, *p* = 0.002). Results were robust to sensitivity analyses, although there was evidence of unbalanced pleiotropy, as assessed by the MR‐Egger intercept test, for the associations between LA and LTL and between BMI and years of schooling (Supporting Information Tables S10 and S12). However, removal of pleiotropic variants detected by the MR‐PRESSO outlier test made no changes to the significance or interpretation of the results derived using IVW regression (*β* = 0.026, 95% CI: 0.010 to 0.042, *p* = 0.003 and *β* = −0.135, 95% CI: −0.155 to −0.116, *p* = 3 × 10^−34^ for the associations between LA and LTL and between BMI and years of schooling, respectively).

Finally, we conducted MR studies exploring the associations of CRP, omega‐6 FAs, LA, and educational attainment with LTL, as well as BMI with omega‐6 FAs, LA, and educational attainment, in nonoverlapping population samples. These generated directionally consistent results with those detected in the primary analyses (Supporting Information Tables S13 and S14). The estimated bias and type I error rate for all significant associations derived from overlapping populations were also negligible, and instruments with sample overlap demonstrated high instrument strength variability (average *I* ^2^ = 98.7%, minimum *I* ^2^ = 98%), suggesting little bias for the MR‐Egger estimates in these analyses (Supporting Information Table S8A). MRlap analyses similarly yielded statistically significant and directionally consistent results with the primary IVW estimates for all associations (Supporting Information Table S8B).

### Mediation analyses

We performed mediation analyses using MVMR to decompose the effects of BMI and LDL‐C on LTL, which act directly, and those acting potentially via CRP, omega‐6 FAs, LA, and years of schooling (Supporting Information Table S15, Figure 2C,D). The negative association between BMI and LTL was attenuated after adjustment for CRP, LA, or years of schooling. However, only CRP and LA influenced LTL independently of BMI (BMI‐adjusted CRP β ± SE: 0.030 ± 0.009, *p* = 4 × 10^−4^ and linoleic acid β ± SE: 0.030 ± 0.014, *p* = 0.03). Similarly, the positive association between LDL‐C and LTL was attenuated when adjusted for either omega‐6 FAs or LA. However, neither omega‐6 FAs nor LA influenced LTL independently of LDL‐C. Based on the same analyses, higher CRP and lower LA were estimated to account for ~28.1% and ~9.4%, respectively, of the total detrimental effect of BMI on LTL. A summary of our main findings is shown in Figure 2E.

## DISCUSSION

We have explored the causal relationships of MetS and its component traits with LTL using univariable MR and MVMR. We demonstrated that obesity, as determined by both elevated BMI and higher body fat percentage, was associated with shorter LTL and thus it might accelerate the development of aging‐related diseases. However, contrary to our hypothesis, neither MetS nor the rest of its components were associated with shorter LTL. In fact, LDL‐C was paradoxically associated with longer telomeres. Although these data conflict with the findings of relatively small observational studies [15, 16, 17, 18], they are in agreement with the results of the largest cross‐sectional study that investigated these relationships to date [19]. Two exceptions were the weakly positive associations of systolic and diastolic BP with LTL reported by Bountziouka et al. [19], which we failed to detect herein. The reason for these inconsistencies is probably reverse causality because, in reverse MR analyses, longer LTL was associated with higher BP (Supporting Information Table S9) [20]. These latter analyses also revealed that longer LTL was paradoxically associated with higher WHRadjBMI, elevated TAG, and increased MetS risk. Collectively, these data indicate that the detected associations of BMI and LDL‐C with LTL were not confounded by reverse causality. Additionally, they highlight that telomere shortening might not be a major driver of adipose tissue (AT) cellular senescence and systemic insulin resistance in humans. In this regard, insulin resistance is the central hallmark and it is thought to be the main driver of MetS. Furthermore, accumulating evidence has suggested that MetS results from inadequate subcutaneous AT storage capacity in the face of continuous excess energy intake, leading to ectopic fat deposition in vital organs [31].

### BMI and LTL

The finding that BMI was negatively associated with LTL in MR analyses is in accordance with most cross‐sectional epidemiological data [15, 17, 19, 32]. In the largest cross‐sectional study to date [19], it was demonstrated that one standard deviation (SD) increase in BMI (~5 kg/m^2^) was associated with shorter mean LTL, which was equivalent to ~1 year of age‐related LTL change (~30 base pairs). Based on the annual LTL change reported by the same group, their estimate compares with ~1.7 years of age‐related LTL change per SD of BMI change in our study, which explored the lifelong consequences of adiposity on LTL. Put in a different way, our data suggest that, compared with an age‐matched lean individual (BMI of 22 kg/m^2^), a person with obesity (BMI of 32 kg/m^2^) has a shorter LTL, equivalent to ~3.4 years of age‐related change. Nonetheless, caution is necessary when extrapolating from the data by Bountziouka et al. [19] to our findings because the age range of the Genetic Investigation of AN thropometric Traits (GIANT) cohort (https://portals.broadinstitute.org/collaboration/giant/index.php/GIANT_Cohorts_and_Group) is broader (17–90 years) than that of the UKBB (40–69 years). Similar findings were also obtained by a cross‐sectional meta‐analysis capturing data from 146,114 individuals, which estimated that a ~five‐unit difference in BMI was equivalent to a ~25 LTL base pair difference [32]. Also consistent with our results, Bountziouka et al. [19] found a stronger association between fat mass percentage and LTL than between BMI and LTL, equivalent to ~1.5 years of age‐related LTL change versus 3.2 years herein. Finally, and in agreement with the meta‐analysis by Gielen et al. highlighted earlier [32], we did not identify any sexual dimorphism in the associations between BMI and LTL.

The predominant mechanism through which obesity might shorten LTL is believed to be increased oxidative stress [33]. In animal studies, reactive oxygen species (ROS) production and lipid peroxidation, consequent to ROS exposure, were shown to be increased in AT in response to high‐fat diet‐induced and genetic obesity. Furthermore, increased AT oxidative stress was associated with oxidative DNA damage in the form of both double stranded DNA breaks and telomere shortening, senescence‐like changes, and increased plasma lipid peroxide levels [34, 35, 36]. Notably, these changes occurred early during the evolution of obesity, prior to the development of glucose intolerance and adipocyte insulin resistance [34, 35], and contributed to activation of the p53 pathway, a key effector of the DNA damage response, which led to proinflammatory cytokine expression and AT inflammatory cell infiltration [35]. Chronic low‐grade inflammation, in turn, was shown to promote ROS production, resulting in further DNA damage and telomere dysfunction, thereby setting up a vicious cycle [37]. Consistent with these findings in rodents, epidemiological studies have reported negative associations between elevated levels of markers of both oxidative stress and inflammation, including CRP, oxidized LDL, and interleukin (IL)‐6 and LTL [38]. Adding to these findings, we now show, using MVMR, that higher CRP, a surrogate of systemic inflammation, accounts for approximately one‐third of the total negative association between BMI and LTL. Furthermore, we identified a novel link between obesity and short LTL, namely lower LA levels. In this respect, as well as being a fuel source, PUFAs have multiple biological functions [39]. These include functioning as ligands for transcription factors such as peroxisome proliferator‐activated receptors, which play essential roles in adipogenesis and lipid metabolism, as well as being substrates for the formation of bioactive molecules, including prostaglandins, leukotrienes, and eicosanoids, which modulate inflammation and ROS production. Which of these function(s) causally links higher circulating LA levels to increased LTL should be the focus of future research. Nonetheless, LA was not causally associated with altered CRP levels in MR analyses (our unpublished data). Finally, MR studies in the UKBB revealed that fewer years of schooling, a surrogate for lower socioeconomic status, might be causally associated with shorter LTL [19]. We now extend these findings by demonstrating that lower educational attainment might link higher BMI with shorter LTL.

### LDL‐C and LTL

Observational studies exploring the relationships between lipid traits and LTL reported uniformly positive baseline and longitudinal associations between HDL‐C and LTL [16, 17, 18, 40], whereas some also found negative cross‐sectional associations of both TAG and total cholesterol with LTL [17]. Although conflicting with these earlier results and being paradoxical, based on the positive links between hypercholesterolemia and age‐related diseases such as atherosclerosis, our MR findings causally linking higher LDL‐C with longer LTL are consistent with observational data from the UKBB [19]. That study reported that higher LDL‐C was associated with ~1.04 years of age‐related LTL change, which compares with ~0.96 years change based on our data; however, the age range of the Global Lipids Genetics Consortium (GLGC) cohort (www.lipidgenetics.org) is also broader than that of the UKBB [40]. Mechanistically, higher circulating omega‐6 FAs and LA levels might link LDL‐C to longer LTL, although we were unable to provide support for this premise from mediation analyses, possibly because of the high genetic correlations of omega‐6 FAs and LA with LDL‐C [24]. Consistent results were reported by a cross‐sectional study investigating the relationships of 226 metabolites measured by nuclear magnetic resonance (NMR) spectroscopy in 11,775 individuals with LTL [23]. Potential links between FA metabolism and functions and LTL were discussed earlier. Furthermore, both FAs and cholesterol are known to play key roles in stem and progenitor cell maintenance, e.g., by activating Wnt [41] and Hedgehog [42] signaling. Finally, the mevalonate pathway, which is required for cholesterol synthesis, promotes the production of ubiquinone, which protects membrane components and mitochondrial DNA from ROS‐induced oxidative damage [43]. Again, which of these activities causally links higher circulating LDL‐C to longer LTL should be investigated in future studies.

### Study strengths and limitations

MR depends on three main assumptions: first, that the IVs associate with the risk factor of interest; second, that they share no common cause with the outcome; and finally, that they do not affect the outcome except through the risk factor. In this regard, all of our exposure instruments were robust (mean exposure *F* score range 21–234). Furthermore, we conducted bidirectional MR and Steiger filtering, which did not provide evidence that our findings were confounded by reverse causality. Finally, based on the MR‐Egger intercept test, none of our significant associations was confounded by unbalanced pleiotropy aside for those between LA and LTL, as well as BMI and years of schooling. However, removal of pleiotropic variants made no changes to the significance or interpretation of the results derived using IVW regression. Regarding limitations, we examined mean LTL length, which does not necessarily translate to TL in tissues and cells relevant to the development of MetS. Second, our findings are based on data from a GWAS conducted in individuals of European ancestry. Therefore, our results and conclusions might not extend to other ancestral populations. Third, MVMR reduces statistical power relative to univariable MR [44], and several of the MVMR estimates, especially those with smaller samples, may be underpowered to detect a relationship with LTL (e.g., omega‐6, PUFA; Supporting Information Table S11). Importantly, the directional relationships aligned between univariable MR and MVMR in these instances. However, we emphasize the need for replication when larger samples become available to test the robustness of these MVMR findings. Fourth, and as previously highlighted, the exposure and outcome instruments relating to some of the MR analyses were derived from overlapping populations. As such, they may be biased toward the outcome‐risk factor association. However, this issue is not typically a concern when applying any two‐sample summary data MR method using one‐sample data from a large population, except for the estimates derived from the MR‐Egger sensitivity analysis, which might show some bias and, therefore, should be interpreted with caution [45]. Importantly, instrument strength variability was high for the overlapping exposures, suggesting that the bias in the MR‐Egger estimate may be minimal [26]. Nevertheless, we emphasize caution in interpreting MR‐Egger estimates from analyses with overlapping samples [45]. Additionally, excluding UKBB participants or using summary statistics generated from alternative cohorts yielded directionally consistent IVW estimates. Furthermore, both the estimated bias and type I error rate for all significant associations derived from overlapping populations were negligible. MRlap analyses [27] were also statistically significant and directionally consistent with the primary IVW estimates for all associations conducted in overlapping populations, suggesting that the latter were minimally affected by sample overlap, weak instrument bias, and winner's curse. Finally, the summary statistics for glycemic traits were generated from a relatively small GWAS [21] and explained only a small percentage of the total variance in these traits, which might account for the lack of associations of fasting blood glucose and insulin levels with LTL.

In summary, using univariable and MVMR analyses, we provide evidence that higher BMI and elevated LDL‐C are causally associated with shorter and paradoxically longer LTL, respectively. The association between raised BMI and shorter LTL is partly driven by increased low‐grade systemic inflammation and lower circulating LA levels. Future experimental studies should examine the mechanistic basis for these links because they could lead to therapeutic strategies to ameliorate oxidative stress, telomere attrition, and/or telomere dysfunction. Finally, our results suggest that telomere shortening is not a major consequence of insulin resistance and MetS, which might result in impaired telomere function instead.

## AUTHOR CONTRIBUTIONS

Conceptualization: Constantinos Christodoulides; investigation and writing, review, and editing: all authors.

## CONFLICT OF INTEREST

The authors declared no conflict of interest.

## Supporting information


**DATA S1.** Supporting information.

**TABLE S1.** GWAS summary statistics used in Mendelian randomization study.
**TABLE S2.** Over‐representation of genes mapped to GWAS loci for BMI within sets of genes mapped to loci for other GWAS catalogue traits in FUMA (https://fuma.ctglab.nl).
**TABLE S3.** Over‐representation of genes mapped to GWAS loci for LDL‐C within sets of genes mapped to loci for other GWAS catalogue traits in FUMA (https://fuma.ctglab.nl).
**TABLE S4.** Mendelian randomization estimates of effects of anthropometric, blood pressure, and metabolic traits on leukocyte telomere length (ieu‐b‐4879, sex‐combined).
**TABLE S5.** MR estimates of effects of additional obesity measures on leukocyte telomere length (ieu‐b‐4879, sex‐combined population).
**TABLE S6.** MR estimates of effects of anthropometric, blood pressure, and metabolic measures and leukocyte telomere length in women and men.
**TABLE S7.** Two‐sample MR estimates of associations of BMI and body fat percentage with leukocyte telomere length in the ENGAGE cohort (“ENGAGE_telo_overall_finalrelease.txt”, Codd et al. 2013; PMID: 23535734).
**TABLE S8.** (A) Bias and type 1 error rate for Mendelian randomization from sample overlap in exposure and outcome GWAS. (B) MRlap results.
**TABLE S9.** Mendelian randomization estimates of effects of leukocyte telomere length (ieu‐b‐4879, sex‐combined) on anthropometric, blood pressure, and metabolic traits.
**TABLE S10.** MR estimates of effects of potential mediators on leukocyte telomere length (ieu‐b‐4879, sex‐combined).
**TABLE S11.** Results of multivariate MR analyses to determine whether the effects of omega‐6 fatty acids and linoleic acid on leukocyte telomere length (sex‐combined) are independent of other polyunsaturated fatty acids.
**TABLE S12.** MR estimates of effects of BMI, and LDL‐C on potential mediators (sex‐combined populations).
**TABLE S13.** MR estimates of effects of additional C‐reactive protein, Fatty acid and educational attainment measures on leukocyte telomere length using non‐overlapping GWAS datasets.
**TABLE S14.** MR estimates of effects of BMI on potential mediators using non‐overlapping GWAS datasets (sex‐combined populations).
**TABLE S15.** Results of multivariate MR analyses to estimate the % mediated effects of BMI, LDL‐C, omega‐6 and linoleic acid, on leukocyte telomere length (sex‐combined).
