# Supplementary material for: Investigating the impact of metabolic syndrome traits on telomere length: a Mendelian randomization study
Source: Obesity (Silver Spring). 2023 Jul 6;31(8):2189–98. doi: 10.1002/oby.23810 (PMC10658743; doi:10.1002/oby.23810)
Supplement: Supplementary file 1 — DATA S1. Supporting information. [file OBY-31-2189-s002.docx]

**Supplementary Information**

**Metabolic Syndrome traits and** **telomere length: a Mendelian Randomization study.**

Nellie Y. Loh ^1^, Daniel Rosoff ^1,2^, Raymond Noordam ^3^, and Constantinos Christodoulides ^1, 4^.

^1^ Oxford Centre for Diabetes, Endocrinology and Metabolism, Radcliffe Department of Medicine, University of Oxford, Oxford OX3 7LE, UK.

^2^ National Institute on Alcohol Abuse and Alcoholism, National Institutes of Health, Bethesda, MD, USA

^3^ Department of Internal Medicine, Section of Gerontology and Geriatrics, Leiden University Medical Center, Leiden, The Netherlands.

^4^ NIHR Oxford Biomedical Research Centre, OUH Foundation Trust, Oxford OX3 7LE, UK.

E-mail addresses:

nellie.loh@gtc.ox.ac.uk

daniel.rosoff@linacre.ox.ac.uk

[R.Noordam@lumc.nl](mailto:R.Noordam@lumc.nl)

costas.christodoulides@ocdem.ox.ac.uk

**Supplementary Methods**

We conducted a Mendelian randomization (MR) study to investigate the associations between the metabolic syndrome (MetS) and MetS traits with leukocyte telomere length (LTL) in men and women of European descent. As genetic instruments, we utilized all genome-wide significant (p<5×10^-8^), independent single nucleotide polymorphisms (SNPs) identified in GWAS meta-analyses for anthropometric (body mass index [BMI], and BMI-adjusted waist-to-hip ratio [WHRadjBMI]) [1, 2], glycemic (fasting glucose and insulin) [3], lipid (triglycerides, HDL-C and LDL-C) [4], and blood pressure (systolic and diastolic BP; [5], ukb-b-20175, ukb-b-7992, https://gwas.mrcieu.ac.uk/datasets/) traits, and MetS as a binary trait [6], conducted in Europeans, pruned for LD using ld_clump, with the default set at LD r^2^<0.001 and a genetic distance of 10Mb (**Table S1**). In the case of fasting insulin, due to the small number of sex-specific GWAS-significant SNPs identified (3 for women and 2 for men), we used as genetic instruments the 19 SNPs established for fasting insulin by MAGIC [3]. Where GWAS summary statistics for MetS traits were not publicly available, genetic instruments were obtained from sex-combined and sex-specific GWAS we conducted in the UKBB (**Table S1**). In brief, GWAS were performed using linear mixed models implemented in BOLT_LMM (version 2.3.2) [7]. We adjusted the analyses for age (and sex, for sex-combined CRP GWAS), and the first 10 genetic principal components and corrected for the genetic correlation matrix which corrects for familial relationships in the UKBB population. Analyses were done on the autosomal chromosomes only. SNPs with a minor allele frequency <0.01, and those with an imputation quality <0.3 were excluded from the generated summary-level data. To obtain a set of independent SNPs for each trait as genetic instruments for MR analyses, we selected all variants with P_BOLT_LMM <5×10^-8^ and performed LD clumping using ld_clump, with the default set at LD r^2^<0.001 and a genetic distance of 10Mb.

As outcome data for the sex-combined analyses, we used the largest publicly available summary-level data for LTL, from a recently conducted GWAS in the UKBB (n = 472,174 participants, ieu-b-4879) [8], and for the sex-specific studies, we used summary-level data from newly conducted GWASs, also in the UKBB. These newly performed analyses were conducted using the same analysis pipeline described above implemented in the BOLT_LMM statistical software package.

To examine reverse causality, we conducted MR study using exposure instruments extracted from the LTL GWAS using extract_instruments() in the TwoSampleMR package (v0.5.6), and outcome data using GWAS summary statistics for MetS and MetS traits from the above mentioned studies.

We used FUMA (<https://fuma.ctglab.nl/>) [9] to identify potential mechanisms whereby BMI, and LDL-C affect LTL. We used the SNP2GENE function of FUMA to translate GWAS signals for BMI (GCST009004) and LDL-C (GCST002222) into sets of mapped genes. The latter were derived based on positional gene mapping and eQTL mapping, using the default settings in FUMA, apart from performing eQTL gene mapping using all the tissues in GTEx and selecting all gene types. We then used the GENE2FUNCTION tool in FUMA, which performs hypergeometric tests to test if genes of interest were over-represented in predefined pathways, in this instance reported genes from the GWAS catalogue ([www.ebi.ac.uk](http://www.ebi.ac.uk)/gwas/). Finally, we cross-referenced the GENE2FUNCTION gene set output with traits and indices of healthy behavior shown to be cross-sectionally associated with LTL in published studies [10, 11]. The latter are highlighted in **Tables S2-3** and were taken forward for further MR analyses, including mediation studies with multivariable MR (MVMR). MR analyses were performed using publicly available European population-specific GWAS summary statistics for omega-3 and omega-6 fatty acids (FAs) [12], linoleic acid (LA) [12], and years of schooling [13], and in the case of CRP, summary statistics from GWAS we conducted in the UKBB (**Table S1**).

Finally, in cases where exposure and outcome data were derived from the same or overlapping populations, additional MR analyses were performed using European population-specific summary data from independent cohorts (**Table S1**). For MR analysis estimating the effect of BMI on LTL using BMI GWAS summary statistics from the GIANT consortium (n = 171,977 women and 152,893 men of European ancestry) [14] (ieu-a-835) we obtained two sets of BMI instruments: the first comprising GWAS-significant independent variants (n = 69 genetic instruments, Table S1); the second comprising exposure data extracted for the 507 GWAS significant SNPs from the BMI meta-analysis by Yengo et al., [2] (Table S1, ieu-b-40) using extract_outcome_data(), filtered for SNPs with F-statistics >10, and further pruned for LD (LD r^2^ < 0.001, genetic distance = 10Mb) (n = 254 genetic instruments). To further test the robustness of the BMI-LTL relationship, we obtained BMI data from the Million Veterans Program (MVP) (n = 215,734 participants of European ancestry) [15], and similarly constructed two sets of BMI instruments: one using all variants with F-statistics >10, the conventional threshold used to determine instrument strength [16], and another using only BMI variants with p < 5x10^-8^ in the MVP summary statistics.

We used the IVW approach for MR analyses [17]. The IVW MR estimate assumes that all instruments included in the analyses are valid, affect the outcome only through the exposure, and do not associate with any confounders. However, since this is often not the case, we performed sensitivity analyses including the MR-Egger regression, weighted-median estimator, and contamination mixture (Conmix). MR-Egger does not force the regression line through the intercept and is therefore able to test for the presence of directional pleiotropy [18]. The weighted-median estimator assumes that at least 50% of the genetic instruments are valid [19]. Conmix identifies groups of genetic variants with similar causal estimates and performs MR robustly in the presence of invalid instruments [20]. Furthermore, we conducted MR analyses after excluding variants with larger effects on outcome than the exposure trait (Steiger-filtering). Results were corrected for multiple testing with p < 0.0125 (0.05/4) considered significant. This cut-off is based on assessments of 4 classes of MetS traits (anthropometric, glycemic, lipid, and blood pressure). Given the high correlation between some traits, this level of correction provides a balance between rigorous results and avoiding false negatives. A statistically significant IVW result coupled with directionally consistent associations from all 3 sensitivity analyses was considered as sufficient evidence to claim a causal effect.

For exposures that had sample overlap with LTL, we calculated instrument I^2^ to assess the variability in the instrument strength. Previous studies testing two-sample MR methods with overlapping exposure and outcome datasets have shown the two-sample MR methods may be safely used when the performed using large biobank data (e.g., the UK Biobank), except for the MR Egger estimate which may be biased to the observational association [21]. However, the MR Egger estimate potential bias in the Egger estimate may be reduced when the variability in the instrument strength is high (i.e., I^2^ > 0.97) [21]. Therefore, we assessed instrument strength variability for the exposures with sample overlap with LTL. As an additional sensitivity test for our exposure-outcome pairs with sample overlap, we also included the MRlap method, which was recently developed to account for sample overlap (even if exact overlap percentage is unknown) and also assess weak instrument bias and winner’s curse [22]. Mrlap assumes a spike-and-slab genomic architecture and uses cross-trait LD-score regression [23] to account for sample overlap between the exposure and outcome datasets and provide a corrected MR estimate [22]. It tests whether the Mrlap estimates is statistically different than the primary IVW estimate.

Next, given the role of the *FADS* gene region *(FADS1*, *FADS2*, and *FADS3*) in omega-6 FA, and more broadly, PUFA, metabolism [24], we sought to further investigate the whether the FADS region impacted LTL. First, we performed colocalization analyses [25] to assess whether there was evidence for a shared causal variant between omega-6 fatty acid levels and LTL in the FADS region. We also explored whether whole blood gene expression of the FADS genes colocalized with LTL. For these analyses, we extracted all variants located $\pm$100 kilobases (and minor allele frequency >0.1) of the FADS region (chromosome 11: 61,560,452-61,659,523) from the omega-6 and linoleic FA GWAS datasets (met-d-Omega_6, met-d-LA, respectively), and also whole blood *FADS1*, *FADS2*, and *FADS3* expression from the eQTLGen consortia (N≤31,684).[26] We performed colocalization analysis using a Python implementation of the Bayesian method *coloc* (v3.2-1) [25], using the *coloc.abf* function with default priors. Using established thresholds, we considered posterior probability (PP.H4) between 0.6 and 0.8 as moderate evidence of colocalization while a posterior probability >0.8 as strong evidence of colocalization [25]. For all colocalization analyses, we failed to find evidence of a shared causal variant between the FADS genes and LTL – Omega-6 PP.H4=0.00359; linoleic acid PP.H4=2.17x10^-3^; *FADS1* PP.H4=0.00227; *FADS2* PP.H4=0.00242; and *FADS3* PP.H4=0.00282.

We also performed cis-instrument MR using independent variants (LD *R^2^ <*0.001*)* associated whole blood *FADS1*, *FADS2*, and *FADS3* expression from the eQTLGen consortia [26] at p<5x10^-8^. We performed MR as described in the Methods section. Results are reported in **Table S5**. FADS variants comprising the instruments were sufficiently strong (i.e., F-statistics >10), and we failed to find evidence of a causal relationship between any of the *FADS* genes and LTL. These colocalization and cis-instrument MR results suggest that omega

MVMR analyses were undertaken only if the primary MR analyses involving the MetS trait, mediator of interest and LTL outcome were significant after Bonferroni correction, with directionally consistent associations in all 3 sensitivity analyses. For this, we used GWAS summary statistics derived from non-overlapping, or, in the case of BMI, partially overlapping, samples for exposure and outcome data. Exposure data for MetS trait and mediator of interest were extracted for MVMR using mv_extract_exposures(). MVMR was subsequently performed to estimate the direct effect of the MetS trait when adjusted for the effect of the mediator and vice versa. In MVMR analyses involving omega-6 FAs and LA UKBB GWAS, the UKBB cohort were partitioned to exclude the 114,999 UKBB participants in the omega-6 FAs and LA GWAS, and new LTL GWAS generated, as above, to minimize bias due to sample overlap.

All MR and MVMR analyses were conducted using the TwoSampleMR (v0.5.6) and MendelianRandomization (v.0.6.0) packages in R (v4.2.1) [27, 28].

**References**

1. Pulit, S.L., et al., *Meta-analysis of genome-wide association studies for body fat distribution in 694 649 individuals of European ancestry.* Hum Mol Genet, 2019. **28**(1): p. 166-174.

2. Yengo, L., et al., *Meta-analysis of genome-wide association studies for height and body mass index in approximately 700000 individuals of European ancestry.* Hum Mol Genet, 2018. **27**(20): p. 3641-3649.

3. Lagou, V., et al., *Sex-dimorphic genetic effects and novel loci for fasting glucose and insulin variability.* Nat Commun, 2021. **12**(1): p. 24.

4. Willer, C.J., et al., *Discovery and refinement of loci associated with lipid levels.* Nat Genet, 2013. **45**(11): p. 1274-1283.

5. Evangelou, E., et al., *Genetic analysis of over 1 million people identifies 535 new loci associated with blood pressure traits.* Nat Genet, 2018. **50**(10): p. 1412-1425.

6. Lind, L., *Genome-Wide Association Study of the Metabolic Syndrome in UK Biobank.* Metab Syndr Relat Disord, 2019. **17**(10): p. 505-511.

7. Loh, P.R., et al., *Efficient Bayesian mixed-model analysis increases association power in large cohorts.* Nat Genet, 2015. **47**(3): p. 284-90.

8. Codd, V., et al., *Polygenic basis and biomedical consequences of telomere length variation.* Nat Genet, 2021. **53**(10): p. 1425-1433.

9. Watanabe, K., et al., *Functional mapping and annotation of genetic associations with FUMA.* Nat Commun, 2017. **8**(1): p. 1826.

10. van der Spek, A., et al., *Fat metabolism is associated with telomere length in six population-based studies.* Hum Mol Genet, 2022. **31**(7): p. 1159-1170.

11. Bountziouka, V., et al., *Modifiable traits, healthy behaviours, and leukocyte telomere length: a population-based study in UK Biobank.* Lancet Healthy Longev, 2022. **3**(5): p. e321-e331.

12. Borges, M.C., et al., *Role of circulating polyunsaturated fatty acids on cardiovascular diseases risk: analysis using Mendelian randomization and fatty acid genetic association data from over 114,000 UK Biobank participants.* BMC Med, 2022. **20**(1): p. 210.

13. Lee, J.J., et al., *Gene discovery and polygenic prediction from a genome-wide association study of educational attainment in 1.1 million individuals.* Nat Genet, 2018. **50**(8): p. 1112-1121.

14. Locke, A.E., et al., *Genetic studies of body mass index yield new insights for obesity biology.* Nature, 2015. **518**(7538): p. 197-206.

15. Huang, J., et al., *Genomics and phenomics of body mass index reveals a complex disease network.* Nat Commun, 2022. **13**(1): p. 7973.

16. Burgess, S., N.M. Davies, and S.G. Thompson, *Bias due to participant overlap in two-sample Mendelian randomization.* Genet Epidemiol, 2016. **40**(7): p. 597-608.

17. Burgess, S., A. Butterworth, and S.G. Thompson, *Mendelian randomization analysis with multiple genetic variants using summarized data.* Genet Epidemiol, 2013. **37**(7): p. 658-65.

18. Bowden, J., G. Davey Smith, and S. Burgess, *Mendelian randomization with invalid instruments: effect estimation and bias detection through Egger regression.* Int J Epidemiol, 2015. **44**(2): p. 512-25.

19. Bowden, J., et al., *Consistent Estimation in Mendelian Randomization with Some Invalid Instruments Using a Weighted Median Estimator.* Genet Epidemiol, 2016. **40**(4): p. 304-14.

20. Burgess, S., et al., *A robust and efficient method for Mendelian randomization with hundreds of genetic variants.* Nat Commun, 2020. **11**(1): p. 376.

21. Minelli, C., et al., *The use of two-sample methods for Mendelian randomization analyses on single large datasets.* Int J Epidemiol, 2021. **50**(5): p. 1651-1659.

22. Mounier, N. and Z. Kutalik, *Bias correction for inverse variance weighting Mendelian randomization.* bioRxiv, 2022: p. 2021.03.26.437168.

23. Bulik-Sullivan, B.K., et al., *LD Score regression distinguishes confounding from polygenicity in genome-wide association studies.* Nature Genetics, 2015. **47**(3): p. 291-295.

24. Mathias, R.A., et al., *FADS genetic variants and omega-6 polyunsaturated fatty acid metabolism in a homogeneous island population.* J Lipid Res, 2010. **51**(9): p. 2766-74.

25. Giambartolomei, C., et al., *Bayesian test for colocalisation between pairs of genetic association studies using summary statistics.* PLoS Genet, 2014. **10**(5): p. e1004383.

26. Võsa, U., et al., *Large-scale cis- and trans-eQTL analyses identify thousands of genetic loci and polygenic scores that regulate blood gene expression.* Nat Genet, 2021. **53**(9): p. 1300-1310.

27. Hemani, G., et al., *The MR-Base platform supports systematic causal inference across the human phenome.* Elife, 2018. **7**.

28. Yavorska, O.O. and S. Burgess, *MendelianRandomization: an R package for performing Mendelian randomization analyses using summarized data.* Int J Epidemiol, 2017. **46**(6): p. 1734-1739.


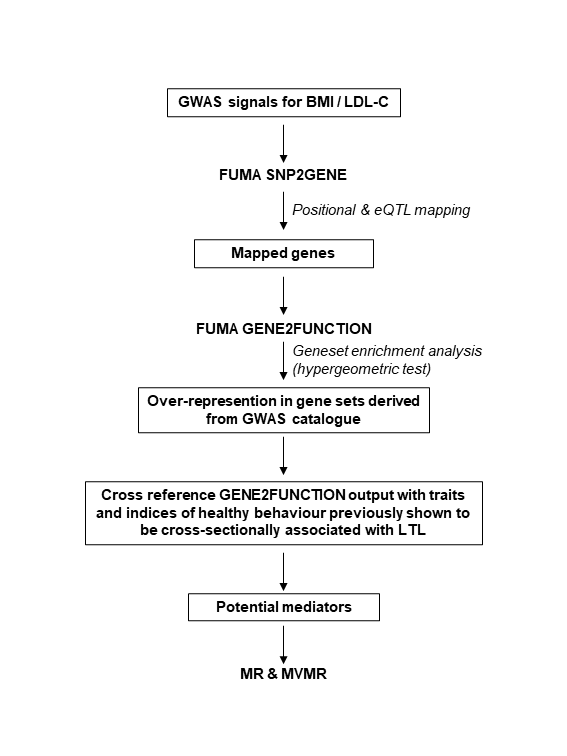


Figure S1. Flow diagram of analyses using FUMA (<https://fuma.ctglab.nl/>) to identify potential mechanisms linking MetS traits and leukocyte telomere length (LTL).

Abbreviations: BMI, body mass index; eQTL, expression quantitative trait loci; LDL-C, low-density lipoprotein cholesterol; MetS, metabolic syndrome; MR, Mendelian randomization; MVMR, multivariable MR.
